# Physical exercise therapy for autism spectrum disorder: a scoping review of systematic reviews

**DOI:** 10.3389/fpubh.2026.1907318

**Published:** 2026-09-03

**Authors:** Fei Fan, Ji Li, Xuan Zhang, Bo Wang, Zilin Chen, Yuchen Hu, Fei Han

**Affiliations:** 1 Department of Pediatrics, Guang’anmen Hospital, China Academy of Chinese Medical Sciences, Beijing, China; 2 Chinese EQUATOR Center, Hong Kong Chinese Medicine Clinical Study Center, Chinese Clinical Trial Registry (Hong Kong), School of Chinese Medicine, Hong Kong Baptist University, Kowloon, Hong Kong SAR, China

**Keywords:** autism Spectrum disorder, motor skills, physical exercise therapy, scoping review, social communication, systematic review

## Abstract

**Background:**

Physical exercise therapy (PET) is used as a non-pharmacological intervention for individuals with autism spectrum disorder (ASD). However, the overall evidence landscape remains unclear.

**Methods:**

This scoping review adhered to the Joanna Briggs Institute methodology and the PRISMA-ScR checklist. Searches were conducted in MEDLINE, EMBASE, Web of Science, and Cochrane Library from database inception to December 4, 2025. Included were systematic reviews that investigated PET for individuals with ASD. Two reviewers independently did data extraction and quality checks (AMSTAR-2).

**Results:**

A total of 50 systematic reviews published between 2013 and 2025 were included. Ball sports/team sports, martial arts, mind–body exercises, aquatic therapy, and structured motor skill interventions were the most frequently studied modalities; the included reviews were generally of low methodological quality; most reviews reported improvements in motor skills, social communication, stereotyped behaviors, executive function, and sleep quality, but results were heterogeneous across outcomes; adverse events were rarely reported.

**Conclusion:**

The available evidence suggests that PET may improve motor, social, behavioral, and sleep outcomes in individuals with ASD, though these findings are limited by low to critically low methodological quality, high heterogeneity, potential publication bias, and a lack of long-term follow-up.

**Systematic review registration:**

The protocol is registered on OSF at DOI: 10.17605/OSF.IO/JVM4H (https://osf.io/jvm4h).

## 1 Introduction

Autism spectrum disorder (ASD) is a neurodevelopmental condition that affects approximately one in every 44 children globally, with a male-to-female ratio of more than four to one (1, 2). The disorder is characterized by persistent deficits in social communication and interaction, alongside restricted, repetitive patterns of behavior, interests, or activities (3). Beyond these core symptoms, individuals with ASD often struggle with other challenges as well-motor coordination difficulties, executive dysfunction, and sleep disturbances, to name a few (4). Multiple factors contribute to the etiology of ASD, ranging from genetic and environmental influences to immune dysregulation (5). More recently, gut microbiota dysbiosis has emerged as another player in the disease process (6).

Physical exercise therapy (PET) has emerged as a popular non-pharmacological option. It is affordable, easy to access, and covers a wide range of activities: aerobic exercises, martial arts, yoga, dance, horse-related activities, water therapy, and organized sports programs (7). In recent decades, a substantial number of systematic reviews and meta-analyses have synthesized the evidence on PET for ASD. However, the results are very mixed. For one thing, systematic reviews exhibit considerable variation in their scope: some reviews focus on one type of exercise, like horseback riding or dance therapy, while others attempt to cover multiple intervention types (8, 9). For another, the outcomes they study are different. Some look at core ASD symptoms, while others look at motor skills or body markers (10). Methodological quality, as measured by the AMSTAR-2 tool, also ranges from critically low to moderate, and many reviews lack preregistered protocols, comprehensive search strategies, or duplicate study selection procedures (7). Finally, even when reviews pose the same research question, they often arrive at conflicting answers. Take social communication as an example: some analyses suggest that physical activity helps, but others fail to find robust effects after adjusting for publication bias (11, 12).

Existing reviews have largely focused on particular outcomes or single intervention modalities (13, 14). A comprehensive synthesis that maps the range of exercise types, study populations, outcome measures, and inconsistencies across the systematic review literature is not yet available. Scoping reviews are designed to map out a broad body of evidence, identify major themes, and highlight research gaps across diverse fields (15). Therefore, we conducted a scoping review of systematic reviews on PET for ASD. Instead of combining effect estimates, this review maps the current evidence, identifies patterns and discrepancies across reviews, and highlights areas where evidence is lacking or unclear.

## 2 Methods

### 2.1 Study design

This scoping review was conducted in accordance with the methodological framework proposed by Arksey and O’Malley (16) and refined by Levac et al. (17), following the Joanna Briggs Institute (JBI) guidelines for scoping reviews. The reporting of this review adheres to the Preferred Reporting Items for Systematic Reviews and Meta-Analyses extension for Scoping Reviews (PRISMA-ScR) checklist (18). The program for this scoping review was registered on the Open Science Framework platform (DOI: 10.17605/OSF.IO/JVM4H), and a detailed protocol has been published *a priori* (19).

### 2.2 Research questions

This scoping review was guided by the following four research questions:

1. What types of exercise modalities have been studied in systematic reviews of PET for individuals with ASD?
2. What population characteristics are primarily addressed in these systematic reviews?
3. What outcome domains are most frequently measured across existing systematic reviews?
4. What are the main features, inconsistencies, and gaps in the current evidence base?

### 2.3 Search strategy

A comprehensive literature search was conducted across four electronic databases: MEDLINE (PubMed), EMBASE, Web of Science, and the Cochrane Library. The search was performed from database inception to December 4, 2025, and was restricted to studies published in English due to resource constraints. The search strategy combined three conceptual blocks: (1) ASD-related terms including “Autism Spectrum Disorder,” “autism,” “ASD,” and “Asperger*”; (2) physical exercise-related terms including “Exercise Therapy,” “physical activity,” “sport*,” “yoga,” “dance,” “horse riding,” and “martial arts”; and (3) systematic review filters including “systematic review” and “meta-analysis.” Boolean operators AND and OR were used to combine search terms. The complete search strategy for MEDLINE (PubMed) is presented in Table 1, and search strategies for all databases are provided in Supplementary file 1.

**Table 1:** Search strategy for MEDLINE (PubMed) database.

Query	Records retrieved
(“Autistic Disorder”[Mesh] OR “Autism Spectrum Disorder” [Mesh] OR autism[tiab] OR asd[tiab] OR autistic[tiab] OR asperger*[tiab]) AND (“Exercise Therapy”[Mesh] OR “Exercise”[Mesh] OR “Sports”[Mesh] OR “Motor Activity”[Mesh] OR exercis*[tiab] OR “physical activity”[tiab] OR “physical activities”[tiab] OR sport*[tiab] OR treadmill[tiab] OR swimming[tiab] OR yoga[tiab] OR “Tai Ji”[tiab] OR Taichi[tiab] OR “martial arts”[tiab] OR dance[tiab] OR cycling[tiab] OR “active play”[tiab] OR “horse riding”[tiab] OR “equestrian therapy”[tiab]) AND (“Meta-Analysis”[Publication Type] OR “Systematic Review”[Publication Type] OR “systematic review”[ti] OR meta-analy*[tiab])	139

### 2.4 Eligibility criteria

Literature that meets the following criteria of the “Population, Intervention, Control Group, Outcome and Study Design” (PICOS) framework can be included (Table 2).

**Table 2:** Eligibility criteria using the PICOS framework.

Eligibility criteria	Description/Characteristics
Population	Individuals with ASD of any age, sex, or ethnicity
Interventions	Must include physical exercise therapy
Comparators	Studies without a comparator will still be eligible for inclusion, as the goal of this scoping review is to map the breadth of available evidence, not to assess comparative efficacy
Outcomes	Core ASD symptoms, comorbidities, quality of life, and physiological indicators (outcomes related to parents or teachers excluded). Core symptoms include social communication deficits and restricted/repetitive behaviors. Physiological indicators may include heart rate variability and body mass index
Study design	Systematic reviews (with or without meta-analysis)

ASD, autism spectrum disorder.

The following types of publications were excluded: (1) duplicate publications; (2) retracted articles; (3) reviews lacking adequate description of methods or results, including protocols, conference abstracts, letters, editorials, and narrative reviews without systematic search; (4) systematic reviews that did not report a quality or risk-of-bias assessment of included primary studies; and (5) non-English-language reviews.

### 2.5 Study selection

All reviewers (FF, JL, FH) were trained on the eligibility criteria before starting the formal review. Inter-rater agreement was assessed using Cohen’s kappa statistic (20) on 15 random publications, with acceptable consistency defined as *κ* > 0.80. Consistency testing was repeated until the threshold was achieved.

**Figure 1:**
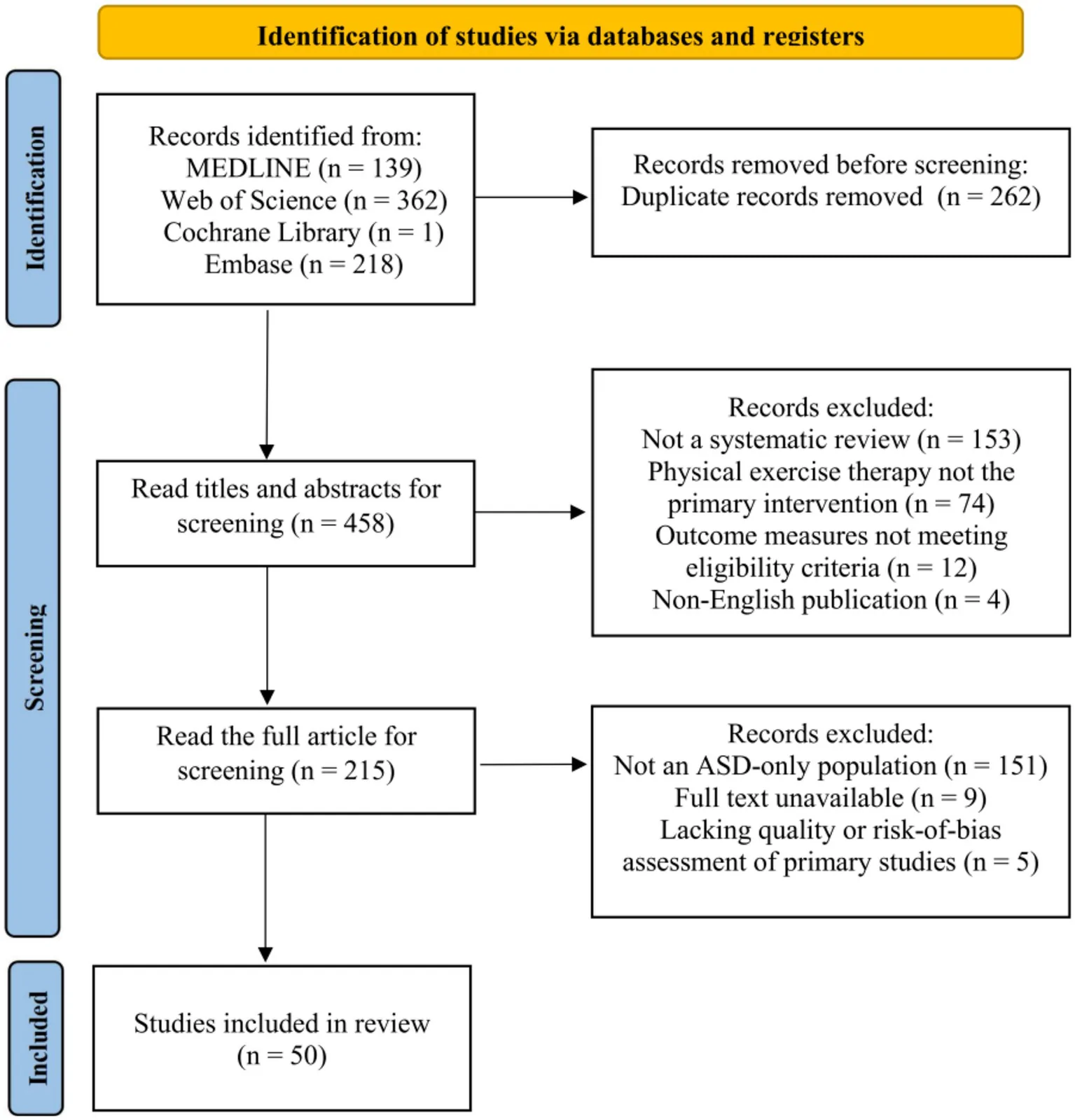
Publication screening process.

All records identified from the search were imported into EndNote X9 (Clarivate Analytics, Philadelphia, PA, United States), and duplicates were removed. Two reviewers (FF, JL) independently screened the titles, abstracts, and keywords of the remaining records based on the eligibility criteria. Records judged as potentially eligible were then retrieved for full-text review. The same two reviewers independently assessed the full-text articles for inclusion, recording reasons for exclusion at each step. Any disagreements during full-text screening were resolved by consensus discussion or by consulting a third reviewer (FH). The search and screening process is summarized using a PRISMA flow diagram (Figure 1).

### 2.6 Data extraction

A standardized data extraction form was developed based on the research questions to extract key data from the included systematic reviews. Data extraction was performed independently by two reviewers (FF, JL) and cross-checked by a third reviewer (FH). The extracted variables included article title, first author, publication year, country, journal, AMSTAR-2 rating, age range, diagnostic criteria, sample size range, co-morbidities, type of exercise, frequency, session duration, total duration (weeks), intervention setting, control type, outcome domains, adverse events, compliance/adherence, original study types, number of original studies, whether meta-analysis was performed, main conclusion, whether inconsistent conclusions were reported, limitations, and future suggestions. Any disagreements during data extraction were resolved through discussion or adjudication by a third reviewer (FH). The same calibration procedure was applied prior to data extraction.

### 2.7 Quality assessment

Methodological quality of the included systematic reviews was assessed independently by two reviewers (FF, JL) using the AMSTAR-2 instrument (21). Any disagreements were resolved by a third reviewer (FH). Based on the 16-item AMSTAR-2 criteria, each review was assigned an overall confidence rating of high, moderate, low, or critically low (21). Quality ratings were not used as exclusion criteria, but greater interpretive weight was given to higher-quality reviews when identifying consistent patterns. Complete AMSTAR-2 item-level assessments for each included review are provided in Supplementary file 2.

### 2.8 Data synthesis

A descriptive qualitative synthesis was conducted following the approach proposed by Arksey and O’Malley (16). Findings were organized according to the four research questions: exercise modalities, population characteristics, outcome domains, and evidence inconsistencies and gaps. In reviews that included both narrative and quantitative data, the results from meta-analyses were given precedence over narrative summaries. When several meta-analyses examined the same outcome, we assessed the direction and magnitude of effects, as well as confidence intervals, highlighting any discrepancies and possible sources of heterogeneity. Due to significant heterogeneity in intervention types, outcome measures, and participant characteristics across reviews, we did not perform quantitative pooling of effect sizes across systematic reviews. Inconsistencies reported within or across reviews were extracted and categorized narratively. Research gaps were identified when review authors explicitly noted insufficient evidence, absence of studies in specific populations, or lack of studies examining particular outcomes. No quantitative pooling of effect sizes across systematic reviews was performed due to substantial heterogeneity in intervention types, outcome measures, and participant characteristics. Our synthesis remains descriptive and exploratory in nature, consistent with JBI guidance for scoping reviews (15). When meta-analyses and narrative summaries were both available for the same outcome, we prioritized meta-analytic findings. To reconcile conflicting meta-analyses, we examined differences in inclusion criteria, intervention types, outcome measures, and statistical approaches and reported the range of effect sizes and confidence intervals rather than selecting a single estimate. Our conclusions were not derived from a vote-counting approach based on the number of reviews reporting positive results. We also explicitly considered heterogeneity and methodological limitations in our interpretive process. Reviews with higher AMSTAR-2 ratings were given greater weight when identifying consistent patterns, though all findings were reported regardless of quality.

## 3 Results

### 3.1 Characteristics of included systematic reviews

A total of 50 systematic reviews published between 2013 and 2025 were included in this scoping review. Among them, 33 reviews (66.0%) included a meta-analysis, while the remaining 17 (34.0%) provided only qualitative or narrative syntheses. The methodological quality of the included reviews, assessed using the AMSTAR-2 tool, was moderate in 17 reviews (34.0%), low in 24 reviews (48.0%), and critically low in 9 reviews (18.0%). Notably, no review was rated as high quality. The most common methodological weaknesses included lack of preregistered protocols, incomplete search strategies, and failure to report funding sources of primary studies. The reviews included in the analysis showed significant differences in both their scope and composition. The number of primary studies within each review varied from 4 to 72. The participant count per individual study typically ranged from 3 to 120 individuals. Regarding the geographic distribution of the included systematic reviews, China led with the highest number of reviews at 48%, followed by the United States at 12%, and then Australia and Spain, each at 6%. Canada, the United Kingdom, and South Korea each account for 4%. The original study types across the 50 systematic reviews predominantly included RCTs, with many reviews also incorporating quasi-experimental designs, controlled clinical trials, pre-post designs, and single-case designs.

### 3.2 Exercise modalities studied

The included systematic reviews examined a wide range of physical exercise modalities, with the most frequently studied categories including ball sports / team sports investigated in 39 reviews (Table 3). Exercise frequencies in the reviewed studies ranged from 1 to 5 sessions per week, with 2-3 sessions/week being most common. Session durations typically ranged from 30 to 90 min, with 45–60 min being the most frequently reported. The total intervention duration across studies ranged from 2 to 48 weeks, with most programs lasting 8–12 weeks. The most frequently reported intervention settings across the systematic reviews were schools, community centers, and clinical/hospital environments, with schools being the most common. Other settings included homes, equestrian centers, outdoor areas, gymnasiums, and university facilities. The most frequently employed control types were passive comparators including waitlist control, no intervention, standard/usual care, and routine activities. Active control conditions—such as barn activities without horseback riding, progressive muscle relaxation, academic education, sedentary games, and low-intensity stretching—were also used to isolate the specific effects of exercise, though many original studies still suffered from high risk of performance and detection bias. The reported frequency, session duration, and total intervention length reflect the most commonly described protocols across the included systematic reviews, not evidence-based optimal prescriptions.

**Table 3:** Exercise modalities and intervention parameters reported across systematic reviews.

Type of exercise	Number of reviews (*n*)	Specific exercise examples
Ball sports/team sports	41	Mini-basketball, basketball, table tennis, soccer/football, Australian rules football, volleyball, floorball, golf, tennis, air volleyball, ball games, recreational ball games, ball playing, ball tapping
Martial arts	34	Karate, kata techniques, taekwondo, judo, mixed martial arts, Chinese mind–body exercise
Mind–body exercises	33	Yoga, Tai Chi, Nei Yang Gong, Chinese mind–body exercise, Qigong, dance movement psychotherapy, creative movement therapies, home-based yoga, structured yoga
Aquatic therapy	30	Swimming, water exercises/hydrotherapy, aquatic training, multisystem aquatic therapy, Halliwick method, water-based training
Structured motor skill interventions	30	SPARK program, fundamental motor skills training, motor skill interventions, physical education programs, balance training, trampoline training, gymnastics, circuit training, sensory integration training, psychomotor training, bilateral training, structured circular training, physical therapy program, ICPL program, motor activity interventions
Equine-assisted/horseback riding	29	Therapeutic horseback riding, hippotherapy, equine-assisted services/activities/therapy, simulated horse-riding programs, equestrianism
Aerobic/conventional physical activities	23	Jogging/running, walking, cycling/stationary biking, aerobic exercise, treadmill walking, high-intensity exercise program, fitness program, stair-stepping, non-static bicycling
Multi-component/combined exercises	17	Combined physical training, multimodal exercise, graded comprehensive practice, coordination + strength exercises, educational exercise programs, combination therapy (exercise + music/social stories), holistic interventions
Exergaming	16	Xbox Kinect, Nintendo Wii, Dance Dance Revolution, Makoto Arena, FroggyBobby, cybercycling, exergaming, virtual reality training, videogame training
Other/Mixed/Unspecified	30	Outdoor adventure programs, dance, sports/play/recreation activities, leisure/recreation programs, group-based organized physical activity, multi-sport camp, rock climbing, skateboarding, roller skating, games, physical games, dog-assisted activities, community-based physical activity, structured physical activity programs

A single systematic review may examine multiple outcome domains; therefore, the sum of *n* exceeds the total number of reviews (*n* = 50).

### 3.3 Population characteristics

Most reviews focused on children and adolescents aged 3 to 17 years, while only one review targeted young adults aged 19–30 years (7). Notably, no systematic review has specifically focused on adults over 30 years of age, highlighting a notable evidence gap for this population. Diagnostic criteria for ASD were most commonly based on DSM-IV/DSM-IV-TR (83%) and DSM-5 (70%), followed by gold-standard observational instruments such as Autism Diagnostic Observation Schedule (ADOS)/ADOS-2 (48%) and standardized rating scales including Gilliam Autism Rating Scale (GARS) (26%) and Childhood Autism Rating Scale (CARS) (22%). Approximately 8% of reviews did not report diagnostic criteria clearly, and a notable number of primary studies within these reviews failed to specify their diagnostic methods. Regarding co-morbidities, approximately 70% of reviews (35/50) provided no information on co-morbidities, the most frequently documented co-morbidities were intellectual disability (8 reviews) and ADHD (6 reviews). The most common intervention environments were schools and community centers, followed by clinical/hospital settings, home, and equestrian centers.

### 3.4 Outcome domains measured

As shown in Table 4, core ASD symptoms were the most frequently assessed outcomes. Specifically, social communication and interaction were examined in 27 reviews, while behavioral symptoms were assessed in 21 reviews. In contrast, quality of life (6 reviews), sleep (5 reviews), and emotional regulation (5 reviews) were less frequently studied, indicating relative evidence gaps. Most studies (47 out of 50) did not report adverse events. Only a few reviews provided specific details of compliance or adherence, such as the use of fidelity checklists, reported dropout rates, categorization based on American College of Sports Medicine (ACSM) guideline adherence, or discussion of behavioral strategies to promote participant compliance.

**Table 4:** Frequency of outcome domains examined across included systematic reviews.

Outcome domain	Specific outcomes measured	Number of reviews (*n*)
Social communication/interaction	Social communication, social cognition, social awareness, social motivation, social functioning, sociability, communication skills	27
Behaviors	Stereotyped/repetitive behaviors, maladaptive behaviors, problem behaviors, hyperactivity, irritability, lethargy, stereotypy, self-injurious behavior	21
Motor skills/balance	Fundamental motor skills, balance, coordination, locomotor skills, object control, stability, motor proficiency	21
Cognitive/executive function	inhibitory control, working memory, cognitive flexibility, planning, attention, memory, academic engagement	16
Physical health outcomes	Cardiorespiratory fitness, muscular strength, flexibility, body composition, BMI, cardio-metabolic health, metabolic profile	10
Quality of life	Quality of life, functional participation, school functioning, daily living participation	6
Sleep	Sleep quality, sleep duration, sleep efficiency, sleep resistance, night awakenings, daytime sleepiness	5
Emotional regulation	Anxiety symptoms, emotional regulation, emotional adaptation, stress	5
Other outcomes	Overall autism severity, autistic traits, on-task behavior, academic responding, school functioning, sensorimotor skills, sensory processing	7

A single systematic review may examine multiple outcome domains; therefore, the sum of *n* exceeds the total number of reviews (*n* = 50).

### 3.5 Main features, inconsistencies, and gaps in the evidence base

Due to the many differences among the systematic reviews, the findings were sorted into three groups: consistent findings, inconsistent or contradictory findings, and evidence gaps. Tables 5–7 show these three groups.

**Table 5:** Consistent findings across systematic reviews.

Outcome domain	Consistent finding	Effect size/condition	Supporting reviews
Motor skills/balance	Exercise interventions produce large to very large improvements in motor skills and balance in children with ASD.	Pooled SMD = 1.07–1.82 for motor/balance; optimal: aquatic training, 2 sessions/week, ≥60 min/session.	Djordjevic et al., 2022 (low); Ji et al., 2023 (low); Jiang et al., 2024 (low); Monteiro et al., 2022 (moderate); Ruggeri et al., 2020 (low); Xing et al., 2025 (critically low) (26–31)
Social functioning	Physical activity interventions significantly improve social communication and social functioning in children and adolescents with ASD.	Small to moderate effect sizes; optimal dose: ~40 sessions, 50 min/session, ≥3 times/week, ≥12 weeks.	Amonkar et al., 2021 (critically low); Bremer et al., 2016 (low); Chan et al., 2020 (low); He et al., 2025 (moderate); Hou et al., 2024 (low); Howells et al., 2019 (low); Jia et al., 2024 (moderate); Jia et al., 2023 (low); Koh et al., 2024 (critically low); Kou et al., 2024 (moderate); Qi et al., 2024 (low); Rosales et al., 2025 (low); Wang et al., 2023 (moderate) (11, 12, 32–42)
Stereotyped/repetitive behaviors	Physical exercise consistently reduces stereotyped and repetitive behaviors in ASD.	Moderate to large effects; higher intensity and longer duration (>12 weeks, >3 times/week) yield greater benefits.	Bremer et al., 2016 (low); Jiang et al., 2024 (low); Li et al., 2024 (moderate); Teh et al., 2022 (low); Wang et al., 2023 (moderate); Yang et al., 2025 (moderate) (26, 37, 42–45)
Executive function	Chronic exercise improves executive function, particularly inhibitory control and cognitive flexibility, but effects on working memory are often non-significant.	Small to moderate effect sizes; effective interventions include mini-basketball, ping pong, martial arts.	Hou et al., 2024 (low); Li et al., 2025 (low); Liang et al., 2022 (critically low); Villodres et al., 2024 (moderate); Wang et al., 2025 (moderate) (46–50)
Sleep	Exercise interventions significantly improve subjective and objective sleep outcomes in children with ASD, including sleep duration, resistance, and efficiency.	Large effects; effective programs last 8–12 weeks.	González-Devesa et al., 2025 (low); Liang et al., 2024 (low); Wang et al., 2025 (moderate) (51–53)

**Table 6:** Inconsistent or contradictory findings across systematic reviews.

Outcome domain	Evidence of inconsistency	Supporting reviews
Social communication/Social functioning	Some reviews found non-significant or attenuated effects after adjusting for publication bias, while others reported significant improvements only in specific subgroups or under certain conditions.	Significant overall effect: He et al., 2025 (moderate); Jia et al., 2024 (moderate); Kou et al., 2024 (moderate); Hou et al., 2024 (low) (social interaction) (33, 34, 38, 41)
		Non-significant or attenuated after adjustment: Chan et al., 2020 (low) (social functioning became non-significant after trim-and-fill); Howells et al., 2019 (low) (communication non-significant) (11, 12)
Stereotyped/repetitive behaviors	Results varied by measurement tool and intervention type. Certain scales showed significant improvements while others did not, and specific exercise modalities failed to produce significant effects in several meta-analyses.	Significant effect (certain measures/subgroups): Jiang et al., 2024 (low) (GARS-2 significant); Li et al., 2024 (moderate) (ball games effective); Yang et al., 2025 (moderate) (ball sports, multi-component effective) (26, 43, 45)
		Non-significant effect (other measures/modalities): Jiang et al., 2024 (low) (RBS-R non-significant); Li et al., 2024 (moderate) (martial arts, water sports, dance non-significant); Yang et al., 2025 (moderate) (equestrian, water sports non-significant); Chen et al., 2022 (low) (stereotypy, irritability, inappropriate speech non-significant) (26, 43, 45, 54)
Motor skills/balance	While most reviews reported significant improvements, one meta-analysis found a non-significant pooled effect due to high heterogeneity, and few studies examined retention or transfer of motor skills.	Significant effect: Djordjevic et al., 2022 (low); Ji et al., 2023 (low); Jiang et al., 2024 (low); Ruggeri et al., 2020 (low); Xing et al., 2025 (critically low) (26–28, 30, 31)
		Non-significant pooled effect: Monteiro et al., 2022 (moderate) (*p* = 0.12, very large ES but high heterogeneity) (29)
Executive function/cognition	Working memory consistently failed to show significant improvement across multiple reviews, while inhibitory control and cognitive flexibility showed positive effects	Significant effect (inhibitory control, cognitive flexibility): Hou et al., 2024 (Low); Liang et al., 2022 (critically low); Villodres et al., 2024 (moderate); Wang et al., 2025 (moderate) (46, 48–50)
		Non-significant (working memory): Liang et al., 2022 (critically low); Li et al., 2025 (low); Wang et al., 2025 (moderate); Wu et al., 2024 (low) (47, 48, 50, 55)
		Non-significant (cognitive domain alone): Rosales et al., 2025 (low) (SSMD = 0.22, *p* = 0.18)
		Intervention-specific: Hou et al., 2024 (low) (stationary bicycle no significant improvement) (46)

**Table 7:** Evidence gaps identified across systematic reviews.

Gap category	Specific gap description	Supporting evidence
Poor methodological quality of primary studies	Most primary studies had small sample sizes, lack of control groups, inadequate blinding and randomization, resulting in low or critically low evidence quality.	Amonkar et al., 2021 (critically low); Bremer et al., 2016 (low); Chan et al., 2020 (low); Howells et al., 2019 (low) (only 3 of 11 studies rated low risk of bias); Ruggeri et al., 2020 (low); Shahane et al., 2024 (moderate) (moderate-to-high risk of bias in 18/22 studies); Wu et al., 2024 (low) (only 10.7% of articles rated high quality) (7, 11, 12, 30, 36, 37, 55)
High heterogeneity across studies	Wide variability in participant characteristics (age, IQ, ASD severity), intervention types, dosages, and outcome measures, making meta-analysis and comparison difficult.	Bodnar et al., 2020 (Critically low); Cai et al., 2025 (low); Chan et al., 2020 (low); (*I*^2^ = 65–74%); Djordjevic et al., 2022 (low) (*I*^2^ = 79.6%); González-Devesa et al., 2025 (low) (*I*^2^ = 97%); Liang et al., 2024 (low) (*I*^2^ = 89–93%); Monteiro et al., 2022 (moderate) (*I*^2^ = 94%) (8, 10, 12, 27, 29, 51, 52)
Small sample sizes	Most primary studies had small sample sizes, leading to low statistical power and imprecise effect estimates.	Bremer et al., 2016 (low); Bodnar et al., 2020 (critically low) (mean sample size 16); Cai et al., 2025 (low); Fang et al., 2019 (moderate); Mortimer et al., 2014 (critically low); Noureen A, 2022 (critically low); Suárez-Manzano et al., 2024 (moderate); Xing et al., 2025 (critically low) (8, 10, 31, 37, 56–59)
Lack of long-term follow-up	Very few studies assessed maintenance of effects beyond immediate post-intervention, limiting understanding of sustainability.	Bremer et al., 2016 (low) (only 6 studies had follow-up, mostly ≤1 month); Amonkar et al., 2021 (critically low); Bodnar et al., 2020 (critically low); He et al., 2025 (moderate); Howells et al., 2019 (low); Ji et al., 2023 (low); Yang et al., 2025 (moderate); Xing et al., 2025 (critically low) (only 8/57 studies reported retention/transfer)
Insufficient reporting of exercise dosage and intensity	Many studies did not measure or report exercise intensity, frequency, or duration, preventing dose–response analysis and replication.	Bremer et al., 2016 (low); Bodnar et al., 2020 (critically low); Chan et al., 2020 (low); Jiang et al., 2024 (low); Liang et al., 2022 (critically low) (only 5 studies reported intensity); Noureen A, 2022 (critically low); Pan et al., 2025 (low); Wu et al., 2024 (low) (only 4 studies provided intensity details); Zhang et al., 2025 (moderate) (8, 12, 14, 26, 37, 48, 55, 58, 60)
Limited inclusion of diverse populations and outcomes	Underrepresentation of females, adults, individuals with intellectual disability, and non-Chinese samples	Amonkar et al., 2021 (critically low); Bodnar et al., 2020 (critically low); Charlier et al., 2025 (moderate) (gender imbalance); González-Devesa et al., 2025 (low) (mini-basketball all from China); Riis et al., 2025 (low) (most studies in children); Shahane et al., 2024 (moderate) (limited young adult data); Wu et al., 2024 (low) (majority male, China-biased) (7, 8, 36, 55, 61, 62)

## 4 Discussion

### 4.1 Overview of PET for ASD

This scoping review mapped 50 systematic reviews examining PET for individuals with ASD. The findings indicate that PET has been studied across a wide range of modalities, with ball/team sports, martial arts, mind–body exercises, aquatic therapy, and structured motor skill interventions being the most frequently investigated. These interventions are predominantly delivered in school or community settings, with common parameters including 2-3 sessions per week, 45–60 min per session, and total intervention durations of 8–12 weeks. When examined by intervention type, ball sports/team sports and aquatic therapy were frequently associated with improvements in motor skills and social outcomes, while mind–body exercises were more commonly linked to executive function and emotional regulation. These observations stem from indirect comparisons across heterogeneous studies, and dose–response relationships have not been formally examined.

**Figure 2:**
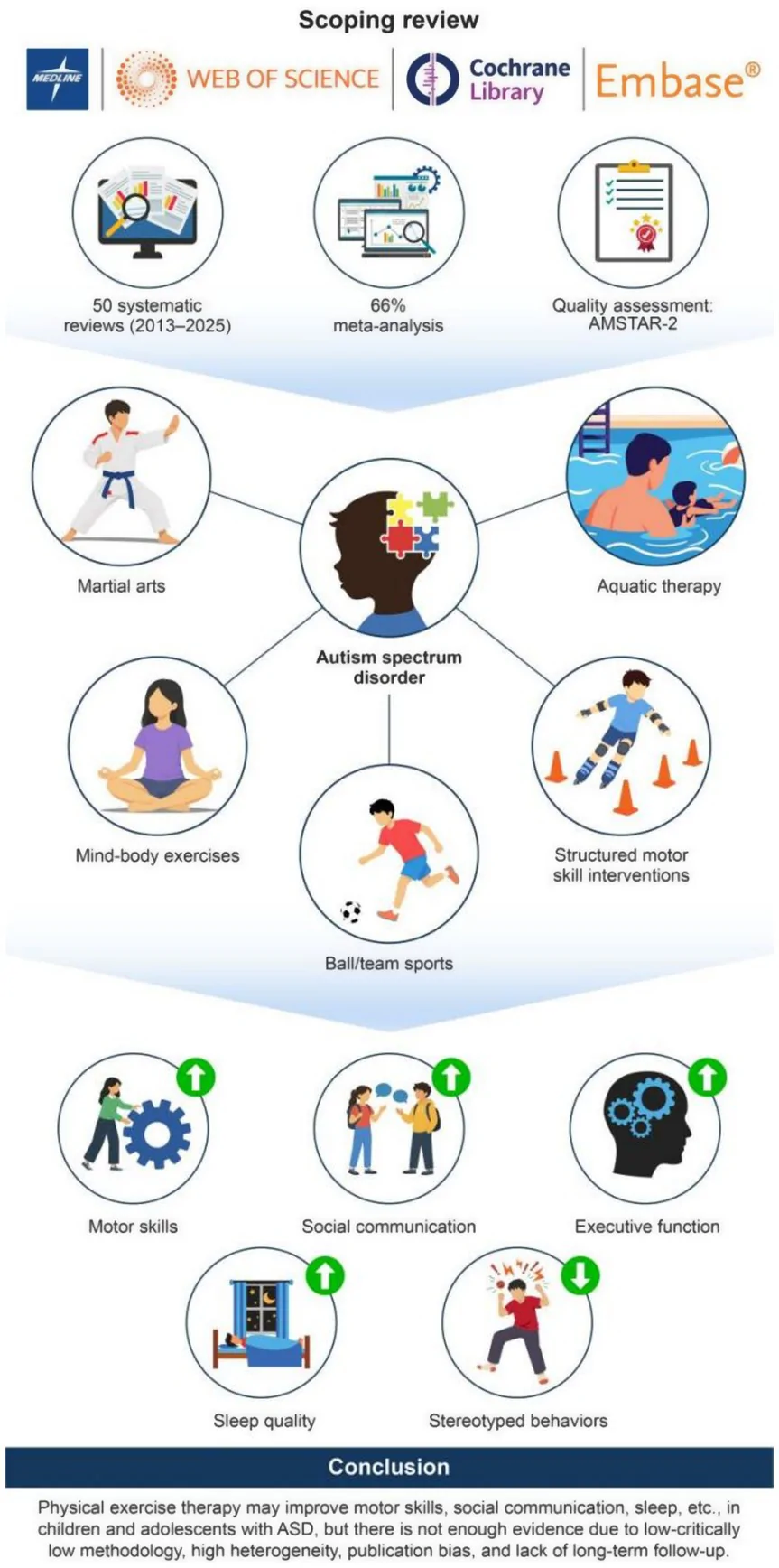
Beneficial effects of PET on ASD core symptoms and comorbidities. The schematic diagram summarizes the consistent beneficial effects of PET on core symptoms (social communication, stereotyped behaviors) and common comorbidities (motor impairment, executive dysfunction, sleep problems) in children and adolescents with ASD based on the included systematic reviews. PET, physical exercise therapy; ASD, autism spectrum disorder.

Consistent evidence across reviews suggests that PET is associated with improvements in motor skills, social communication, stereotyped behaviors, executive function (particularly inhibitory control), and sleep quality in children and adolescents with ASD. These findings align with the broader literature on physical activity benefits in neurodevelopmental populations (22). However, the evidence is predominantly focused on pediatric groups, with very little inclusion of adults or young adults. This age disparity merits emphasis. While our eligibility criteria did not exclude adults, the near absence of systematic reviews in this population points to a substantive evidence gap that constrains the generalizability of current findings. Several mechanisms could explain the benefits of PET in ASD. Repetitive, structured exercise is thought to drive neuroplastic changes in brain regions that underlie motor control, social cognition, and executive function, including the cerebellum, prefrontal cortex, and mirror neuron system (23). Exercise-related improvements in inhibitory control and cognitive flexibility may promote behavioral regulation and social adaptation (24). The sensory-motor integration inherent in aquatic therapy, martial arts, and yoga may also aid sensory processing in ASD (25). These interpretations remain speculative, however, and await verification in neuroimaging and biomarker studies. (Figure 2).

### 4.2 Methodological quality and heterogeneity

A primary concern is that, according to the AMSTAR-2 tool evaluation, none of the reviews achieved a high-quality rating. Only 34.0% were deemed to be of moderate quality, while a significant 66.0% fell into the low or very low-quality categories. Frequent issues identified include the absence of pre-registered protocols, incomplete search strategies, failure to disclose the funding sources of the primary studies, and a lack of bias risk assessment in many of the primary studies. These methodological deficiencies compromise the reliability of the overall effect estimation. Moreover, there are considerable discrepancies in participant characteristics (such as age, IQ, and autism severity), intervention types and doses, assessment indicators, and control conditions across various reviews. The pronounced heterogeneity, along with the publication bias noted in some reviews, imply that the perceived impact of PET on key outcomes might have been overstated. Another concern is that our synthesis may be subject to vote-counting bias. The frequency of positive findings does not necessarily reflect the strength of the underlying evidence, particularly given the overlapping scopes of many reviews and the likelihood of publication bias. Beyond methodological quality, heterogeneity across studies complicates interpretation. The included reviews varied widely in participant characteristics, intervention protocols, outcome measures, and study designs. These sources of heterogeneity limited comparability across reviews, complicated meta-analytic pooling, and constrained generalizability. Thus, although some positive findings were consistent, heterogeneity remains a major obstacle to definitive conclusions.

### 4.3 Evidence gaps and future directions

The original studies present several critical deficiencies, such as the absence of essential evidence. Firstly, many of these studies are plagued by issues such as small sample sizes, inadequate control groups, and ineffective blinding and randomization, which collectively result in reduced statistical power. Furthermore, there is a notable absence of long-term follow-up data, as most studies only evaluate outcomes immediately following the intervention, thereby limiting the assessment of long-term effectiveness. Additionally, the reporting of exercise intensity, frequency, and duration is insufficient, making dose–response analysis unfeasible. The studies also lack adequate representation of women, adults, and non-Chinese populations, which restricts their applicability to these groups. Future research should emphasize the execution of large-scale, meticulously designed randomized controlled trials with appropriate blinding, establish standardized exercise parameters, and incorporate diverse participant groups. Long-term follow-up should be employed to assess the durability of effects. Moreover, the pre-registration of systematic reviews and adherence to reporting guidelines such as PRISMA and AMSTAR-2 should be mandatory to enhance the rigor of research methodologies. Future studies should also directly compare different exercise dosages to establish evidence-based prescription guidelines.

### 4.4 Limitations of this scoping review

This scoping review has several limitations. Methodologically, we restricted our search to English-language systematic reviews, which may have introduced language bias. We also did not directly assess the quality of primary studies within each review, instead relying on the AMSTAR-2 ratings of the reviews themselves. Furthermore, we did not formally quantify overlap among primary studies using approaches such as the Corrected Covered Area. Because many included systematic reviews have overlapping scopes and inclusion criteria, the apparent consistency across multiple reviews may partly reflect repeated inclusion of the same primary studies rather than independent corroboration. No GRADE assessment was performed, and substantial heterogeneity across reviews precluded quantitative synthesis or meta-analysis of effect sizes.

Concerning the underlying evidence base, most primary studies were short-term, single-site trials with small sample sizes, limiting external validity. Publication bias is likely, as small or null findings are less frequently published, which may have inflated the apparent effects. Adverse events were rarely reported, with only three reviews mentioning them.

In terms of generalizability, the evidence is geographically skewed-nearly half of the reviews originated from China. Most focused on children and adolescents, with underrepresentation of females, adults over 30, and individuals with intellectual disability. Long-term follow-up data were scarce, precluding assessment of sustainability. Finally, by confining our scope to systematic reviews that assessed the quality of primary studies, we may have omitted narrative reviews that could offer supplementary insights. Collectively, these factors constrain the generalizability and reliability of our conclusions.

## 5 Conclusion

This scoping review suggests that PET may be associated with improvements in motor skills, social communication, repetitive behaviors, executive functions, and sleep quality. Nevertheless, the current evidence is constrained by low to critically low methodological quality, considerable heterogeneity, publication bias, and a lack of long-term follow-up data, and our review serves to map the evidence landscape rather than to establish definitive effectiveness. Key evidence gaps include the absence of studies that represent adults and females, along with a lack of detailed descriptions of specific exercise methods. Future research should focus on conducting large-scale RCTs, using blinding, standardizing and unifying exercise methods, incorporating a wider range of participants, and extending follow-up periods to assess long-term effectiveness.

## Data Availability

The datasets used and analyzed during the current study are available from the corresponding author on reasonable request.

## References

[1] Wang L Wang B Wu C Wang J Sun M. Autism Spectrum disorder: neurodevelopmental risk factors, biological mechanism, and precision therapy. Int J Mol Sci. (2023) 24:1819. doi: 10.3390/ijms24031819, 36768153 PMC9915249

[2] Tafolla M Singer H Lord C. Autism spectrum disorder across the lifespan. Annu Rev Clin Psychol. (2025) 21:193–220. doi: 10.1146/annurev-clinpsy-081423-031110, 39836874

[3] Green RM Travers AM Howe Y McDougle CJ. Women and autism spectrum disorder: diagnosis and implications for treatment of adolescents and adults. Curr Psychiatry Rep. (2019) 21:22. doi: 10.1007/s11920-019-1006-3, 30852705

[4] Malwane MI Nguyen EB Trejo S Kim EY Cucalón-Calderón JR. A delayed diagnosis of autism spectrum disorder in the setting of complex attention deficit hyperactivity disorder. Cureus. (2022) 14:e25825. doi: 10.7759/cureus.25825, 35836458 PMC9273190

[5] Xiong N Yu Z. Autism spectrum disorder in the genomic era: a comprehensive review of etiology, precision diagnostics, clinical outcomes, and emerging gene-editing therapies. Neuropsychiatr Dis Treat. (2026) 22:585060. doi: 10.2147/NDT.S585060, 41908337 PMC13022905

[6] Zhang S Han F Wang Q Fan F. Probiotics and prebiotics in the treatment of autism spectrum disorder: a narrative review. J Integr Neurosci. (2024) 23:20. doi: 10.31083/j.jin2301020, 38287844

[7] Shahane V Kilyk A Srinivasan SM. Effects of physical activity and exercise-based interventions in young adults with autism spectrum disorder: a systematic review. Autism. (2024) 28:276–300. doi: 10.1177/13623613231169058, 37128159

[8] Bodnar I Pavlova I Khamade A. Physical education of children with autism spectrum disorders: a systematic review of structure and effects of interventional programs. Physiother Q. (2020) 28:61–70. doi: 10.5114/PQ.2020.96232

[9] Aithal S Moula Z Karkou V Karaminis T Powell J Makris S. A systematic review of the contribution of dance movement psychotherapy towards the well-being of children with autism spectrum disorders. Front Psychol. (2021) 12:719673–3. doi: 10.3389/fpsyg.2021.719673, 34744883 PMC8564751

[10] Cai B Miao Y Zhao J Ying X Lin W. The effect of exercise intervention on cognitive function and quality of life with autism spectrum disorder: a systematic review and meta-analysis. Act. Espanol. Psiquiatr. (2025) 53:839–56. doi: 10.62641/aep.v53i4.2040, 40791034 PMC12353249

[11] Howells K Sivaratnam C May T Lindor E McGillivray J Rinehart N. Efficacy of group-based organised physical activity participation for social outcomes in children with autism spectrum disorder: a systematic review and meta-analysis. J Autism Dev Disord. (2019) 49:3290–308. doi: 10.1007/s10803-019-04050-9, 31102193

[12] Chan JS Deng K Yan JH. The effectiveness of physical activity interventions on communication and social functioning in autistic children and adolescents: a meta-analysis of controlled trials. Autism. (2021) 25:874–86. doi: 10.1177/1362361320977645, 33307759

[13] Gao X Xu G Fu N Wang L Ben Q Shen M . Exercise interventions for health outcomes in children with autism spectrum disorder: an umbrella review of meta-analyses of clinical trials. Neurosci Biobehav Rev. (2025) 173:106144. doi: 10.1016/j.neubiorev.2025.106144, 40254115

[14] Zhang L Zhang C Yuan X Ji Y. The impact of exercise interventions on core symptoms of 3–12-year-old children with autism spectrum disorder: a systematic review and network meta-analysis. Eur Child Adolesc Psychiatry. (2025) 34:1991–2005. doi: 10.1007/s00787-025-02696-8, 40131458

[15] Munn Z Peters MDJ Stern C Tufanaru C McArthur A Aromataris E. Systematic review or scoping review? Guidance for authors when choosing between a systematic or scoping review approach. BMC Med Res Methodol. (2018) 18:143. doi: 10.1186/s12874-018-0611-x, 30453902 PMC6245623

[16] Arksey H O’Malley L. Scoping studies: towards a methodological framework. Int J Soc Res Methodol. (2005) 8:19–32. doi: 10.1080/1364557032000119616

[17] Levac D Colquhoun H O’Brien KK. Scoping studies: advancing the methodology. Implement Sci. (2010) 5:69. doi: 10.1186/1748-5908-5-69, 20854677 PMC2954944

[18] Tricco AC Lillie E Zarin W O’Brien KK Colquhoun H Levac D . PRISMA extension for scoping reviews (PRISMA-ScR): checklist and explanation. Ann Intern Med. (2018) 169:467–73. doi: 10.7326/M18-0850, 30178033

[19] Fan F Li J Zhang X Chen Z Hu Y Han F. Physical exercise therapy for autism spectrum disorder: protocol for a scoping review of systematic reviews. Syst Rev. (2026) 15:200. doi: 10.1186/s13643-026-03193-y, 42071249 PMC13352702

[20] Nasser M. Cochrane handbook for systematic reviews of interventions. Am J Public Health. (2020) 110:753–4. doi: 10.2105/ajph.2020.305609

[21] Shea BJ Reeves BC Wells G Thuku M Hamel C Moran J . AMSTAR 2: a critical appraisal tool for systematic reviews that include randomised or non-randomised studies of healthcare interventions, or both. BMJ. (2017) 358:j4008. doi: 10.1136/bmj.j4008, 28935701 PMC5833365

[22] Carcelén-Fraile MDC Hita-Contreras F Mesas-Aróstegui MA Aibar-Almazán A. Effects of physical activity on executive function and emotional regulation in children and adolescents with neurodevelopmental disorders: a systematic review and meta-analysis. Healthcare. (2025) 13:2415. doi: 10.3390/healthcare13192415, 41095504 PMC12524881

[23] Zhang W Cai K Xiong X Zhu L Sun Z Yang S . Alterations of triple network dynamic connectivity and repetitive behaviors after mini-basketball training program in children with autism spectrum disorder. Sci Rep. (2025) 15:2629. doi: 10.1038/s41598-025-87248-5, 39838077 PMC11751186

[24] Song X Hou Y Lei Z Zhou S. Multilevel meta-analysis of the effect of exercise intervention on inhibitory control in children with ASD. Front Psychol. (2025) 16:1632555. doi: 10.3389/fpsyg.2025.1632555, 41143048 PMC12546240

[25] Homayounnia Firouzjah M Majidi Yaeichi N Hematinia R. The effectiveness of sensory-motor integration exercises on social skills and motor performance in children with autism. J Autism Dev Disord. (2024) 55:1902–9. doi: 10.1007/s10803-024-06325-2, 38565778

[26] Jiang J Wang G Gu Q Wang X Liu J. Quantifying the efficacy of physical activity on motor skills and stereotypies in children with autism spectrum disorder: a meta-analysis of randomized controlled trials from the last decade. Res Autism Spectr Disord. (2024) 114:102395. doi: 10.1016/j.rasd.2024.102395

[27] Djordjević M Memisevic H Potic S Djuric U. Exercise-based interventions aimed at improving balance in children with autism spectrum disorder: a meta-analysis. Percept Mot Skills. (2022) 129:90–119. doi: 10.1177/00315125211060231, 34936828

[28] Ji Y-Q Tian H Zheng Z-Y Ye Z-Y Ye Q. Effectiveness of exercise intervention on improving fundamental motor skills in children with autism spectrum disorder: a systematic review and meta-analysis. Front Psych. (2023) 14:1132074. doi: 10.3389/fpsyt.2023.1132074, 37377477 PMC10291092

[29] Monteiro CE Da Silva E Sodre R Costa F Trindade AS Bunn P . The effect of physical activity on motor skills of children with autism spectrum disorder: a meta-analysis. Int J Environ Res Public Health. (2022) 19:14081. doi: 10.3390/ijerph192114081 36360956 PMC9655847

[30] Ruggeri A Dancel A Johnson R Sargent B. The effect of motor and physical activity intervention on motor outcomes of children with autism spectrum disorder: a systematic review. Autism. (2020) 24:544–68. doi: 10.1177/1362361319885215, 31782658

[31] Xing Y Wu X. Effects of motor skills and physical activity interventions on motor development in children with autism spectrum disorder: a systematic review. Healthcare. (2025) 13:489. doi: 10.3390/healthcare13050489, 40077051 PMC11899540

[32] Qi K Wang X Xu Q Hu B Wang Z Bialas M. Effect of physical activity on social communication impairments in children with autism spectrum disorder: a meta-analysis. Heliyon. (2024) 10:e39053. doi: 10.1016/j.heliyon.2024.e39053, 39640832 PMC11620107

[33] He J Gong Y Yin M Zhang L Wu X. Optimal dosage of group-based organized physical activity for enhancing social abilities in autistic children: insights from a multilevel meta-analysis. Int J Behav Nutr Phys Act. (2025) 22:87. doi: 10.1186/s12966-025-01787-8, 40597379 PMC12210644

[34] Hou Y Song Z Deng J Song X. The impact of exercise intervention on social interaction in children with autism: a network meta-analysis. Front Public Health. (2024) 12:1399642. doi: 10.3389/fpubh.2024.1399642, 39206007 PMC11349572

[35] Rosales MR Butera CD Wilson RB Zhou J Maus E Zhao H . Systematic review and meta-analysis of the effect of motor intervention on cognition, communication, and social interaction in children with autism spectrum disorder. Phys Occup Ther Pediatr. (2025) 45:688–710. doi: 10.1080/01942638.2025.2498357, 40320371

[36] Amonkar N Su W-C Bhat AN Srinivasan SM. Effects of creative movement therapies on social communication, behavioral-affective, sensorimotor, cognitive, and functional participation skills of individuals with autism spectrum disorder: a systematic review. Front Psych. (2021) 12:722874. doi: 10.3389/fpsyt.2021.722874, 34867515 PMC8637167

[37] Bremer E Crozier M Lloyd M. A systematic review of the behavioural outcomes following exercise interventions for children and youth with autism spectrum disorder. Autism. (2016) 20:899–915. doi: 10.1177/1362361315616002, 26823546

[38] Jia M Zhang J Pan J Hu F Zhu Z. Benefits of exercise for children and adolescents with autism spectrum disorder: a systematic review and meta-analysis. Front Psych. (2024) 15:1462601. doi: 10.3389/fpsyt.2024.1462601, 39435130 PMC11491325

[39] Jia S Guo C Li S Zhou X Wang X Wang Q. The effect of physical exercise on disordered social communication in individuals with autism spectrum disorder: a systematic review and meta-analysis of randomized controlled trials. Front Pediatr. (2023) 11:1193648. doi: 10.3389/fped.2023.1193648, 37456563 PMC10347521

[40] Koh SH. Analyzing the influence of physical exercise interventions on social skills in children with autism spectrum disorder: insights from meta-analysis. Front Psychol. (2024) 15:1399902. doi: 10.3389/fpsyg.2024.1399902, 39421839 PMC11484040

[41] Kou R Li Z Li M Zhou R Zhu F Ruan W . Comparative effectiveness of physical exercise interventions on sociability and communication in children and adolescents with autism: a systematic review and network meta-analysis. BMC Psychol. (2024) 12:712. doi: 10.1186/s40359-024-02210-w, 39614353 PMC11607877

[42] Wang S Chen D Yang Y Zhu L Xiong X Chen A. Effectiveness of physical activity interventions for core symptoms of autism spectrum disorder: a systematic review and meta-analysis. Autism Res. (2023) 16:1811–24. doi: 10.1002/aur.3004, 37539450

[43] Li L Jia S Wang P Li S Wang X Zhu X. A network meta-analysis of the effect of physical exercise on core symptoms in patients with autism spectrum disorders. Front Neurol. (2024) 15:1360434. doi: 10.3389/fneur.2024.1360434, 38784898 PMC11113547

[44] Teh EJ Vijayakumar R Tan TXJ Yap MJ. Effects of physical exercise interventions on stereotyped motor behaviours in children with ASD: a meta-analysis. J Autism Dev Disord. (2022) 52:2934–57. doi: 10.1007/s10803-021-05152-z, 34236592

[45] Yang J Li R. Systematic review and randomized controlled trial meta-analysis of the effects of physical activity interventions and their components on repetitive stereotyped behaviors in patients with autism spectrum disorder. Front Psychol. (2025) 16:1579345. doi: 10.3389/fpsyg.2025.1579345, 40486887 PMC12141328

[46] Hou Y Wang Y Deng J Song X. Effects of different exercise interventions on executive function in children with autism spectrum disorder: a network meta-analysis. Front Psych. (2024) 15:1440123. doi: 10.3389/fpsyt.2024.1440123, 39345918 PMC11427388

[47] Li H Zhang R. The effect of exercise intervention on balance and executive function in children with autism spectrum disorder: a meta-analysis. BMC Sport. Sci. Med. Rehabil. (2025) 17:80. doi: 10.1186/s13102-025-01142-1, 40217333 PMC11987222

[48] Liang X Li R Wong SHS Sum RKW Wang P Yang B . The effects of exercise interventions on executive functions in children and adolescents with autism spectrum disorder: a systematic review and meta-analysis. Sports Med. (2022) 52:75–88. doi: 10.1007/s40279-021-01545-3, 34468951

[49] Villodres GC Ruiz-Prieto S Muros JJ. Impact of physical activity on executive function in primary school children with autism spectrum disorder: a systematic review. Internat. J. Develop. Disabil. (2024) 72:913–32. doi: 10.1080/20473869.2024.2385637, 42516840 PMC13403461

[50] Wang H Cheng G Li M-m. The effectiveness and sustained effects of exercise therapy to improve executive function in children and adolescents with autism: a systematic review and meta-analysis. Eur J Pediatr. (2025) 184:286. doi: 10.1007/s00431-025-06115-7, 40199800

[51] González-Devesa D Sanchez-Lastra MA Outeda-Monteagudo B Ayán-Pérez C Diz-Gómez JC. The effect of exercise training on sleep quality in autism spectrum condition: systematic review and meta-analysis of randomized controlled trials. Res Autism Spectr Disord. (2025) 119:102516. doi: 10.1016/j.rasd.2024.102516, 42574925

[52] Liang X Haegele JA Tse AC-Y Li M Zhang H Zhao S . The impact of the physical activity intervention on sleep in children and adolescents with autism spectrum disorder: a systematic review and meta-analysis. Sleep Med Rev. (2024) 74:101913. doi: 10.1016/j.smrv.2024.101913, 38442500

[53] Wang J Li J Wang F You Y. Exercise intervention influences on sleep and anxiety in children with autism spectrum disorder: a meta-analyses of randomized controlled trials. Neuropediatrics. (2025) 56:302–9. doi: 10.1055/a-2561-8487, 40107307

[54] Chen S Zhang Y Zhao M Du X Wang Y Liu X. Effects of therapeutic horseback-riding program on social and communication skills in children with autism spectrum disorder: a systematic review and meta-analysis. Int J Environ Res Public Health. (2022) 19:114449. doi: 10.3390/ijerph192114449, 36361327 PMC9655675

[55] Wu Y Ding L Zhang Q Dong Y Tao C Li Z . The effect of physical exercise therapy on autism spectrum disorder: a systematic review and meta-analysis. Psychiatry Res. (2024) 339:116074. doi: 10.1016/j.psychres.2024.116074, 38986177

[56] Fang Q Aiken CA Fang C Pan Z. Effects of exergaming on physical and cognitive functions in individuals with autism spectrum disorder: a systematic review. Games for health journal. (2019) 8:74–84. doi: 10.1089/g4h.2018.0032, 30332294

[57] Mortimer R Privopoulos M Kumar S. The effectiveness of hydrotherapy in the treatment of social and behavioral aspects of children with autism spectrum disorders: a systematic review. J Multidiscip Healthc. (2014) 7:93–104. doi: 10.2147/jmdh.S55345, 24520196 PMC3917923

[58] Noureen A Islam T Rashid MA Kabir MS Mathialagan A Farzana Y . Indoor motor exercise regimens improve social cognition in children with autism spectrum disorder: a systematic review. J Pharmaceut Negative Result. (2022) 13:2020–8. doi: 10.47750/pnr.2022.13.S03.298

[59] Suarez-Manzano S Ruiz-Ariza A de Loureiro NEM Martinez-Lopez EJ. Effects of physical activity on cognition, behavior, and motor skills in youth with autism spectrum disorder: a systematic review of intervention studies. Behav Sci. (2024) 14:330. doi: 10.3390/bs14040330, 38667126 PMC11047543

[60] Pan K Ye W Wu Z. Physical exercise interventions improve muscular strength, cardiorespiratory fitness, body composition and flexibility in children and adolescents with autism spectrum disorder: a systematic review and meta-analysis. Res Autism. (2025) 127:202668. doi: 10.1016/j.reia.2025.202668

[61] Charlier L Cordeiro L Neto JLC Signini DF Barbosa-Silva J Corbellini C . Effects of physical exercise on cardiometabolic health in individuals with autism spectrum disorder: a systematic review. Healthcare. (2025) 13:439. doi: 10.3390/healthcare13040439, 39997314 PMC11855590

[62] Riis K Samulski B Neely KA Laverdure P. Physical activity for anxiety for autistic people: a systematic review. J Autism Dev Disord. (2025) 55:2663–79. doi: 10.1007/s10803-024-06356-9, 38755488 PMC12296984

