# Supplementary material for: Physical exercise therapy for autism spectrum disorder: a scoping review of systematic reviews
Source: Front Public Health. 2026 Sep 3;14:1907318. doi: 10.3389/fpubh.2026.1907318 (PMC13582335; doi:10.3389/fpubh.2026.1907318)
Supplement: Supplementary file 1 [file Supplementary_file_1.docx]

| **Database** | **Query** | **Records retrieved** |
| --- | --- | --- |
| **MEDLINE** | ("Autism Spectrum Disorder"[Mesh] OR autism[tiab] OR asd[tiab] OR autistic[tiab] OR asperger*[tiab] OR "high functioning autism"[tiab] OR "high-functioning autism"[tiab]) AND ("Exercise Therapy"[Mesh:exp] OR "Exercise"[Mesh:exp] OR "Sports"[Mesh:exp] OR "Motor Activity"[Mesh:exp] OR "Physical Fitness"[Mesh] OR exercis*[tiab] OR physical activit*[tiab] OR sport*[tiab] OR treadmill[tiab] OR swimming[tiab] OR yoga[tiab] OR "Tai Ji"[tiab] OR Taichi[tiab] OR "martial arts"[tiab] OR dance[tiab] OR cycling[tiab] OR "active play"[tiab] OR "horse riding"[tiab] OR "equestrian therapy"[tiab]) AND ("Meta-Analysis"[Publication Type] OR "Systematic Review"[Publication Type] OR "systematic review"[ti] OR meta-analy*[tiab]) | 139 |
| **Embase** | ('autism spectrum disorder'/de OR autism:ti,ab,kw OR asd:ti,ab,kw OR autistic:ti,ab,kw) AND ('kinesiotherapy'/exp OR 'exercise'/de OR 'physical exercise'/exp OR 'yoga'/de OR 'tai chi'/de OR 'swimming'/de OR 'dance therapy'/de OR 'hippotherapy'/de OR exercis*:ti,ab,kw OR yoga:ti,ab,kw OR 'tai ji':ti,ab OR taichi:ti,ab OR swim*:ti,ab,kw OR dance:ti,ab,kw OR 'horse riding':ti,ab,kw OR 'equestrian therap*':ti,ab,kw OR hippotherapy:ti,ab,kw) AND ('meta analysis'/de OR 'systematic review'/de) | 218 |
| **Web of Science** | (autis* OR asd OR "autism spectrum" OR autistic OR asperger* OR "high functioning autism" OR "high-functioning autism") AND (exercis* OR "physical activit*" OR sport* OR treadmill OR swimming OR yoga OR "tai chi" OR taichi OR "martial arts" OR dance OR cycling OR "active play" OR "horse riding" OR "equestrian therap*") AND ("systematic review" OR "meta-analysis" OR "meta analy*") | 362 |
| **Cochrane Library** | #1 (Autism Spectrum Disorder OR autism OR autistic OR asperger* OR ASD OR PDD OR "Pervasive Developmental Disorder" OR "high functioning autism"):ti,ab,kw  #2 (exercis* OR (physical NEXT activit*) OR (motor NEXT skill*) OR sport* OR treadmill OR swimming OR yoga OR (horse NEXT riding) OR (equestrian NEXT therapy) OR (tai NEXT ji) OR taichi OR (martial NEXT arts) OR dance OR dancing OR cycling OR (active NEXT play) OR (physical NEXT education) OR (outdoor NEXT activit*)):ti,ab,kw  #3 #1 AND #2 | 1 |

**Search Strategy**
